# Suitability of target region amplified polymorphism (TRAP) markers to discern genetic variability in sweet sorghum

**DOI:** 10.1186/s43141-020-00071-5

**Published:** 2020-10-06

**Authors:** Yehia A. Khidr, Sileshi A. Mekuriaw, Adel E. Hegazy, Enass Amer

**Affiliations:** 1 Department of Plant Biotechnology, Genetic Engineering and Biotechnology Research Institute, University of Sadat City, Sadat City, Egypt; 2 Department of Biology, Bahir Dar University, Bahir Dar, Ethiopia

**Keywords:** Lignin, Sucrose, Molecular markers

## Abstract

**Background:**

Sweet sorghum is an emerging biofuel candidate crop with multiple benefits as a source of biomass energy. Increase of biomass and sugar productivity and quality is a central goal in its improvement. Target region amplified polymorphism (TRAP) is a polymerase chain reaction (PCR) based functional marker system that can detect genetic diversity in the functional region of target genes. Thirty sweet sorghum genotypes were used to study the potential of 24 pairs of TRAP marker system in assessing genetic diversity with regard to three lignin and three sucrose biosynthesis genes.

**Results:**

A total of 1638 bands were produced out of which 1161 (70.88%) were polymorphic at least at one locus. The average polymorphic information content (PIC), resolving power (RP), marker index (MI), Shannon’s diversity index (H), and gene diversity values were 0.32, 8.86, 1.74, 3.25, and 0.329, respectively. Analysis of molecular variance (AMOVA) revealed a highly significant genetic variation both within and among accessions studied (*P* = 0.01). However, the variation within the population was higher than among the populations (accessions). Bootstrap analysis showed that the number of loci amplified using this marker system is sufficient to estimate the available genetic diversity. The thirty genotypes were categorized into five clusters using a similarity matrix at 0.72 coefficient of similarity. The genotypes were also grouped mostly according to their geographic origin where the Ethiopian and Egyptian genotypes tend to fall in specific clusters. Moreover, the genotypes reflected the same pattern of distribution when ordinated using principal coordinate analysis.

**Conclusions:**

In conclusion, TRAP marker can be used as a powerful tool to study genetic diversity in sweet sorghum.

## Background

Sweet sorghum (*Sorghum bicolor* L. Moench) is a member of the family *Poaceae* and genus *Sorghum* (2*n* = 20). The genus *Sorghum* encompasses three species: *Sorghum bicolor*, *Sorghum propinquum*, and *Sorghum halepense*. The species *S. bicolor* includes three subspecies: *S. bicolor* subsp. *bicolor*, *S. bicolor* subsp. *drummondii*, and *S. bicolor* subsp. *verticilliflorum* [1]. Sweet sorghum is a variety of sorghum known for its long, sweet, and juicy stalk. Due to its high biomass production and sugar accumulation potential under low input conditions and unique stress tolerance capability, it appears as an important candidate bioenergy crop [2, 3]. It has a potential to produce as much as 13.2 t/ha of total sugars that is equivalent to 8000 (l/ha)/year of ethanol depending on the genotype, environment, and input conditions [4].

Global energy consumption is predicted to rise nearly by 50% between 2018 and 2050 according to the US energy administration (www.eia.gov/ieo). On the other hand, depletion of fossil resources, global warming due to the increase in atmospheric carbon load and soaring oil prices are posing eminent challenges on the global economy and environmental safety [5]. Biofuels such as ethanol produced from plant biomass with lower food-feed tradeoffs and input requirements are among the alternatives to avert this challenge.

Lignocellulosic biomass obtained from agricultural waste and grass species like sweet sorghum is a promising source of nonfood-based biofuel feedstocks. Lignin is a complex phenylpropanoid polymer that binds the cell walls of supporting and conductive tissues, such as fibers and tracheary elements hindering the efficient bioconversion of structural sugars into ethanol requiring a harsh rate-limiting pretreatment procedure [6–8].

Lowering lignin content and enhancing sugar accumulation potential are the main breeding objectives for the improvement of the crop [9, 10]. Decreasing of the recalcitrance of biomass for fermentation can be achieved through downregulation of enzymes in the monolignol biosynthetic pathway [11–13]. This calls for a targeted breeding effort to optimize biomass yield and quality. Understanding the degree and pattern of genetic diversity existing in the available germplasm resources is a key step in the breeding process geared toward addressing the gap.

An array of marker systems, including randomly amplified polymorphic DNA (RAPD) [14, 15], amplified fragment length polymorphism (AFLP) [16, 17], inter simple sequence repeats (ISSR) [15], simple sequence repeats (SSR) [17–21], and single nucleotide polymorphisms (SNP) [18, 22] markers have been used to study patterns of genetic diversity and relationship among sweet sorghum accessions and breeding lines. Markers that are designed to measure genetic diversity for target-specific breeding purposes should be based on functionally characterized genes since they may reflect functional polymorphisms [23]. Unlike conventional random markers such as RAPD, restriction fragment length polymorphism (RFLP), AFLP, and SSR functional diversity markers that are physically associated with coding regions of the genome are developed by designing primers from annotated expressed sequence tag (EST) sequences of specific gene [23, 24].

Target region amplification polymorphism (TRAP) is a polymerase chain reaction (PCR) based marker system involving a fixed primer designed from EST sequence data of a target gene combined with an arbitrary primer having an AT- or CG-rich core sequenc e[25]. It is a multi-locus marker that can potentially be exploited for genotyping and tagging candidate genes controlling a trait of interest. These markers are characterized by their simplicity, high yield, reproducibility, and can be sequenced [26]. TRAP marker systems are used to study genetic diversity in different crop species such as castor bean [27], guarana [28], lettuce [29], mango [30], salvia [31], sugarcane [32, 33], sunflower [34], and wheat [35].

A variety of marker attributes including the percentage of polymorphic bands (PPB), polymorphic information content (PIC), Shannon diversity index (H), resolving power (RP), and marker index (MI) are used to estimate the overall efficiency of the marker system. The PIC of a marker evaluates the discriminatory capability of a marker system among genotypes based on their pattern of polymorphism using the frequency and distribution of the alleles in a locus. The MI and RP of a marker are used to estimate the efficiency of a marker system to characterize a larger set of germplasm resources [36]. To the best of our knowledge, there is no report on the utilization of TRAP marker system on sweet sorghum as a functional marker targeting important traits of interest. This study is designed to evaluate the efficiency of TRAP markers based on three lignin pathway and three sucrose metabolism genes to study genetic variability and relatedness among sweet sorghum genotypes.

## Methods

### Plant material

A panel of 30 sweet sorghum materials comprising seventeen landrace collections and three cultivars from Ethiopia and thirteen elite cultivars obtained from the sugar crops research institute, Agriculture Research Center, Egypt, were planted in pots for genomic DNA extraction for about 15 days (Table 1). The Ethiopian landrace collections with their passport data were obtained from the Ethiopian Biodiversity Institute (EBI), Addis Ababa, Ethiopia.

**Table 1:** List and source of 30 sweet sorghum genotypes used in the study

No.	Accession	Type	Source	No.	Accession/cultivar	Type	Source
1	acct1_1SW	Land race	Ethiopia	16	acct39_1 ST	Land race	Ethiopia
2	acct31SW	Land race	Ethiopia	17	acct24_1ST	Land race	Ethiopia
3	acct6_1 SW	Land race	Ethiopia	18	Mn1383	Cultivar	Egypt
4	acct264 ST	Land race	Ethiopia	19	Ramada	Cultivar	Egypt
5	acct221 ST	Land race	Ethiopia	20	GKGaba	Cultivar	Egypt
6	acct133 SW	Land race	Ethiopia	21	Mn1500	Cultivar	Egypt
7	acct133_1 SW	Land race	Ethiopia	22	Umbrella	Cultivar	Egypt
8	acct301ST	Land race	Ethiopia	23	Honey	Cultivar	Egypt
9	acct1_1SW	Land race	Ethiopia	24	Brandes	Cultivar	Egypt
10	acct32 SW	Land race	Ethiopia	25	Eg-b	Cultivar	Egypt
11	acct41 SW	Land race	Ethiopia	26	Eg-a	Cultivar	Egypt
12	acct10_1 NW	Land race	Ethiopia	27	Eg-c	Cultivar	Egypt
13	acct38_1 ST	Land race	Ethiopia	28	A-2267-2	Cultivar	Ethiopia
14	acct31SW	Land race	Ethiopia	29	NGTJ-2	Cultivar	Ethiopia
15	acct12_2NW	Land race	Ethiopia	30	Karmifma	Cultivar	Ethiopia

### DNA extraction

Genomic DNA was extracted from fresh young leaves of 15 days old sweet sorghum seedlings digested in liquid nitrogen using Thermo Scientific GeneJET Plant Genomic Extraction mini kit according to the manufacturers protocol. The DNA concentrations and quality were determined using Thermo Scientific™ NanoDrop™ 2000 spectrophotometer. All the DNA samples were adjusted to a concentration of 10 ng/μL using Thermo Scientific nuclease-free water for PCR amplification.

### Primer design

A total of twenty-four primer combinations comprising six specific and four arbitrary primers were used in this study (Table 2). The gene-specific (fixed) primers were designed from the sorghum expressed sequence tags (ESTs) database from the NCBI (https://www.ncbi.nlm.nih.gov/gene). Primer3 software tool (http://www.bioinformatics.nl/cgi-bin/primer3plus/primer3plus.cgi) was used by setting maximum and minimum size set to 18 and 20 base pairs and the optimum, maximum, and minimum Tm were set to 53 °C, 55 °C, and 50 °C, respectively. Four arbitrary primers that comprised three selective nucleotides at the 3′-end, 4 AT/GC rich sequences targeting intron-exon regions, respectively, and 11 random (filler) sequences at the 5′-end [37].

**Table 2:** Fixed and arbitrary primers used as TRAP markers

	Gene/primer name	Gene ID/accession	Nucleotide sequence (5′ > 3′)	Tm
**Lignin-related genes**	Cinnamoyl coA reductase (CCR)	LOC8054741	GTCAGGAACCCAGATGAC	55
	Cinnamoyl alcohol dehydrogenase (CAD)	LOC110434683	GGGCTTCAAAGTACCCTA	54
	Caffeic acid 3-O-methyltransferase (COMT)	LOC8070884	CAAGAAGCTCCTCGAGTT	54
**Sucrose-related genes**	Sucrose synthase (susy)	Sb01g033060	ATGGTATTCTCCGCAAGTGG	58
	Soluble acid invertase (Inv)	Sb04g000620	CATCGTTGCAGGGTATCCC	59
	Sucrose phosphate synthase (sps)	Sb05g007310	GCAAACCTTACGCTGATACTG	56
**Arbitrary primer**	Em_1_		GACTGCGTACGAATTTGC	49
	Em_2_		GACTGCGTACGAATTGAC	53
	Em_3_		GACTGCGTACGAATTTGA	53
	Em_4_		GACTGCGTACGAATTAAT	52

### PCR amplification

PCR amplification was conducted with a final reaction volume of 15 μL: 7.5 master mix (COSMO PCR RED) manufactured by Willowfort, UK, 3.5 μL (35 ng) of template DNA, 0.5 μL of forward and reverse primers (10 mM each), and 3 μL nuclease-free water. The PCR was carried out with an initial denaturation at 94 °C for 5 min and 94 °C for 45 s; annealing at 35 °C for 45 s and extension at 72 °C for 1 min, for the first 5 cycles followed by 35 cycles with annealing temperatures ranging from 50-55 °C depending on the primer _Tm_ and a final extension step at 72 °C for 7 min using Techne TC-4000 Thermal Cycler. The PCR products were resolved using 1.5% agarose gel in a 0.5X Tris borate-EDTA (TBE) as a running buffer and stained with ethidium bromide (0.5 μg/mL). The size of the fragments was estimated by visual comparison with a 50-bp DNA ladder (Thermo Scientific). The gel was photographed under UV light using a Bio-Rad gel imaging system.

### Data analysis

The bands were scored manually for each test genotype as presence (1) or absence (0) in a binary data matrix. Only clear and unambiguous bands were scored. The degree of informativeness of the TRAP marker combinations was assessed by analyzing the banding pattern produced by each primer measured in terms of the total number of bands (T), number of polymorphic bands (PL), and their proportion (%P). The suitability of the marker to study the genetic variability of the sweet sorghum genotypes was evaluated by calculating five marker attributes namely polymorphic information content (PIC), resolving power (RP), effective multiplex ratio (EMR), marker index (MI), and Shannon diversity index (H) for each marker combination and averaged for all the marker combinations. The PIC was computed using allele frequencies generated as a ratio of amplified fragments to the total number of genotypes as *PIC* = 2*f*_*i*_(1 − *f*_*i*_) where “fi” is the frequency an amplified allele “i” while (1 − *f*_*i*_) is the frequency of the null allele [37]. The marker index was computed as the product of effective multiplex ratio (EMR) and diversity index (DI), where EMR refers to the number of polymorphic markers generated per assay and the DI is the average PIC value [38]. The RP indicates the ability of the markers to distinguish between accessions [36] for each primer combinations was computed as: *RP* =  ∑ *Ib*, where “Ib” is the band in formativeness that takes the values of 1 − [2×| 0.5 − *P*_*i*_| ], where “*P*_*i*_” is the proportion of each genotype containing the band [39]. The diversity index, which indicates the genetic diversity of the germplasm, was calculated using the formula $DI=1-1/L\sum {P}_i^2$ in which “*P*_*i*_” is the allele frequency (each individual allele was considered unique) and “L” is the number of loci. Shannon’s diversity index (H) was computed using the formula: *H* =  −  ∑ *f*_*i*_ ln *f*_*i*_where “fi” is the frequency of an amplified for a marker band relative to all bands amplified across all the genotypes. The expected heterozygosity or gene diversity that refers to the probability that two randomly chosen alleles from the population are different and was computed using the PowerMarker V3.0 software [40]. Pairwise Nei’s unbiased genetic distance was calculated and the resulting matrix was used for cluster analysis. Cluster analysis was performed using the unweighted pair group method with arithmetic mean (UPGMA) and a phylogenetic tree was constructed using the NTSYS-pc 2.10 software [41]. Bootstrap analysis was performed to determine the number of loci enough to effectively estimate the genetic diversity existing among the genotypes using the GENES software [42]. The pairwise genetic distance matrix utilized above for cluster analysis was used for the analysis. A simulated matrix was established by resampling using 50 to 1000 numbers of loci with 10,000 permutations. The simulated distance matrices were then correlated with the actual distance matrix and verified using Kruskal stress value [43]. The number of loci that can sufficiently estimate the genetic diversity in a set of genotypes corresponds to a value where the Kruskal stress value (*E*) less than 0.05 and the correlation coefficient (*r*) approaches the maximum value (1). The underlying genetic relationships among the 30 sweet sorghum genotypes were further analyzed using DARwin® software version (6.0.21) which is based on distance multivariate exploratory analysis (dissimilarity matrix). The analysis of molecular variance (AMOVA) was computed to determine the proportions of the molecular variance between and within groups of accessions (GenAlex 6 in Microsoft Excel).

## Results

**Fig. 1:**
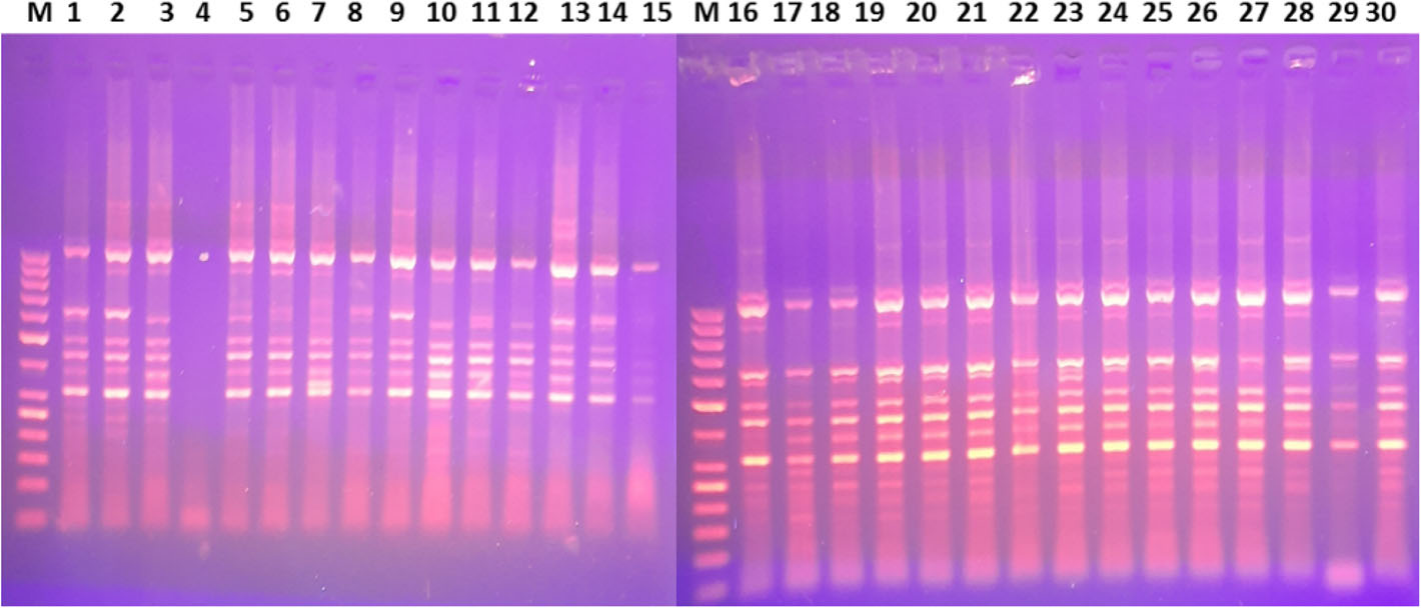
TRAP profiling of thirty sweet sorghum genotypes amplified with COMT/Em2 primer combination in a 1.5% Agarose gel compared with a 50 bp lane

A total of 1638 bands were amplified from the 30 sweet sorghum genotypes using 24-marker combinations. Out of these bands, 1161 (70.88%) were polymorphic at least at one of the loci. The number of fragments generated per primer pair ranged from 36 (SUSy/Em03) to 108 (COMT/Em01) with a mean of 68.25 bands per primer combination (Fig. 1). A relatively higher number of bands were amplified using the lignin-based markers (927) with 71.18% polymorphism as compared to markers based on sucrose metabolism genes (711) with 69.62% polymorphism.

The maximum percentage of polymorphic loci was 87.5% (CCR/Em04) while the minimum percent polymorphism 55.56% (CAD/Em02). The percent polymorphism for markers based on lignin related genes ranged between 55.56 and 87.5%, while the range for their sucrose-based counterparts was 60 to 80% (Table 3). The polymorphic information content (PIC) computed as a mean over all the genotypes ranged from 0.22 (SPS/EM04) to 0.45 (SPS/EM01) for sucrose related markers and 0.18 (CCR/EM04) to 0.42 (CCR/EM03) for lignin related markers (Table 4). The overall average PIC displayed by all marker combinations was 0.32 that showed moderate to higher informativeness of the marker. Both groups of TRAP marker systems designed based on sucrose and lignin related genes showed almost similar PIC value (0.32 and 0.33), respectively.

**Table 3:** Polymorphism of 24 TRAP marker combinations on 30 sweet sorghum genotypes

Gene function	Primer (marker) combinations		Total number of bands	Number of polymorphic bands	Polymorphic bands (%)
	Forward (fixed)	Reverse (arbitrary)			
Lignin biosynthesis	**Cinnamoyl coA reductase (CCR)**	Em01	54	36	66.67
		Em02	63	36	57.14
		Em03	63	45	71.43
		Em04	72	63	87.50
	**Cinnamoyl alcohol dehydrogenase (CAD)**	Em01	90	63	70.00
		Em02	81	45	55.56
		Em03	63	36	57.14
		Em04	63	36	57.14
	**Caffeic acid 3-O-methyltransferase (COMT)**	Em01	108	90	83.33
		Em02	99	81	81.82
		Em03	81	63	77.78
		Em04	90	72	80.00
Total			**927**	**666**	
Mean			**142.62**	**102.46**	**70.46**
Sucrose biosynthesis	**Sucrose synthase (SUSy)**	Em01	45	36	80.00
		Em02	54	36	66.67
		Em03	36	27	75.00
		Em04	45	27	60.00
	**Sucrose phosphate synthase (SPS)**	Em01	72	54	75.00
		Em02	90	54	60.00
		Em03	45	27	60.00
		Em04	63	45	71.43
	**Soluble acid invertase (Inv)**	Em01	72	54	75.00
		Em02	54	36	66.67
		Em03	81	63	77.78
		Em04	54	36	66.67
Total			**711**	**495**	
Mean			**59.25**	**41.25**	**69.52**
Grand total			**1638**	**1161**	
Grand mean			**68.25**	**48.38**	**70.88**

*T* total number of loci; *PL* polymorphic loci; *P%* polymorphism percentage

**Table 4:** TRAP marker attributes and diversity indices of 30 sweet sorghum genotypes

Gene function	Primer (marker) combinations		PIC	RP	MI	H	G D
	Forward (fixed)	Reverse (arbitrary)					
**Lignin biosynthesis**	**Cinnamoyl coA reductase (CCR)**	Em01	0.24	6.53	0.96	3.38	0.214
		Em02	0.29	6.87	1.16	3.34	0.344
		Em03	0.42	8.13	2.10	3.17	0.281
		Em04	0.18	10.67	1.26	3.37	0.202
	**Cinnamoyl alcohol dehydrogenase (CAD)**	Em01	0.37	12.27	2.59	3.19	0.415
		Em02	0.38	7.27	1.90	3.38	0.343
		Em03	0.39	5.40	1.56	3.28	0.340
		Em04	0.35	6.27	1.40	3.27	0.374
	**Caffeic acid 3-O-methyltransferase (COMT)**	Em01	0.31	17.80	3.10	3.37	0.317
		Em02	0.23	13.73	2.07	3.36	0.200
		Em03	0.41	10.53	2.87	3.28	0.393
		Em04	0.37	14.20	2.96	3.10	0.324
**Mean**			**0.33**	**9.97**	**1.99**	**3.29**	**0.374**
**Sucrose Biosynthesis**	**Sucrose synthase (SUSy)**	Em01	0.27	6.53	1.08	3.10	0.368
		Em02	0.27	6.67	1.08	3.28	0.290
		Em03	0.29	4.80	0.87	3.37	0.381
		Em04	0.23	5.27	0.69	3.37	0.233
	**Sucrose phosphate synthase (SPS)**	Em01	0.45	9.93	2.70	2.15	0.397
		Em02	0.39	9.73	2.34	3.31	0.322
		Em03	0.33	5.40	0.99	3.39	0.481
		Em04	0.22	9.07	1.10	3.34	0.258
	**Soluble acid invertase (Inv)**	Em01	0.32	10.93	1.92	3.30	0.248
		Em02	0.37	6.93	1.48	3.29	0.409
		Em03	0.33	10.93	2.31	3.30	0.402
		Em04	0.32	6.73	1.28	3.38	0.357
**Mean**			**0.32**	**7.74**	**1.49**	**3.22**	**0.345**
**Grand mean**			**0.32**	**8.86**	**1.74**	**3.25**	**0.329**

*PIC* Polymorphic information content; *RP* Resolving power; *MI* Marker index; *H* Shannon diversity index, *GD* Gene diversity (expected heterozygosity)

The resolving power of the markers ranged from 5.40 (CAD/EM03) to 17.80 (COMT/EM01) for markers based on lignin-related genes while it ranged from 4.80 (SUSy/EM03) to 10.93 (SuSy/Em3 and COMT/Em1) for markers based on sucrose metabolism genes. The mean resolving power was 8.86 for all markers tested over the genotypes. However, lignin-based markers showed a relatively higher RP (9.97) as compared to sucrose-related markers (7.74). The marker with a higher RP (COMT/Em1) had a higher capability to distinguish among the genotypes. The marker index ranged from 0.69 (SUSy/EM04) to 3.10 (COMT/EM1) with a mean value of 1.74. The lignin-based markers displayed a higher mean marker index (1.99) than their sucrose-based counterparts (1.49).

### Genetic diversity

The mean Shannon’s diversity index of genotypes with respect to the 24 marker combinations was 3.25, indicating a significant genetic variability among the genotypes using this marker system. The lignin and sucrose gene-based markers gave a comparable value (3.29 and 3.22), respectively. The average gene diversity detected using the 24 TRAP markers was 0.329. The gene diversity detected among genotypes using lignin-based markers was higher (0.379) than the value obtained using markers designed for sucrose genes (0.345) (Table 4). Analysis of molecular variance (AMOVA) revealed a highly significant genetic variation both within and among studied accessions (*P* = 0.01). However, the total variation was 79.02% within the accessions while the remaining was 20.98% among the accession (Table 5).

**Table 5:** AMOVA for 30 sweet sorghum accessions using 24 TRAP marker combinations

Source	df	SS*	Est. var.	%D	*P* value*
Among accessions	29	121.712	3.24	79.02 (Fst = 0.724)	0.001
Within accessions	690	23.222	0.86	20.98	
Total	719	144.934	4.10		

*df* Degrees of freedom, *SS* Sum of square, *Est.var.* Estimated variance, *%D* Distribution of total variance

### Bootstrap analysis

Bootstrap analysis was conducted by setting a series of values for the number of loci in each resampling cycle. When the number of loci used for resampling increased, it was observed that the correlation coefficient between the simulated and original distance matrices increased while the Kruskal stress value decreased (Fig. 4). When resampling was performed with 401 loci the correlation coefficient value was 0.86 which is closer to its maximum value (1) and the stress value reached 0.035. A stress value lower than 0.05 is acceptable to estimate the number of loci sufficient to assess genetic diversity [42]. However, in the present study, we have a total of 1638 loci that is by far higher than the number of loci required to estimate the genetic diversity among the test genotypes.

### Cluster analysis

**Fig. 2:**
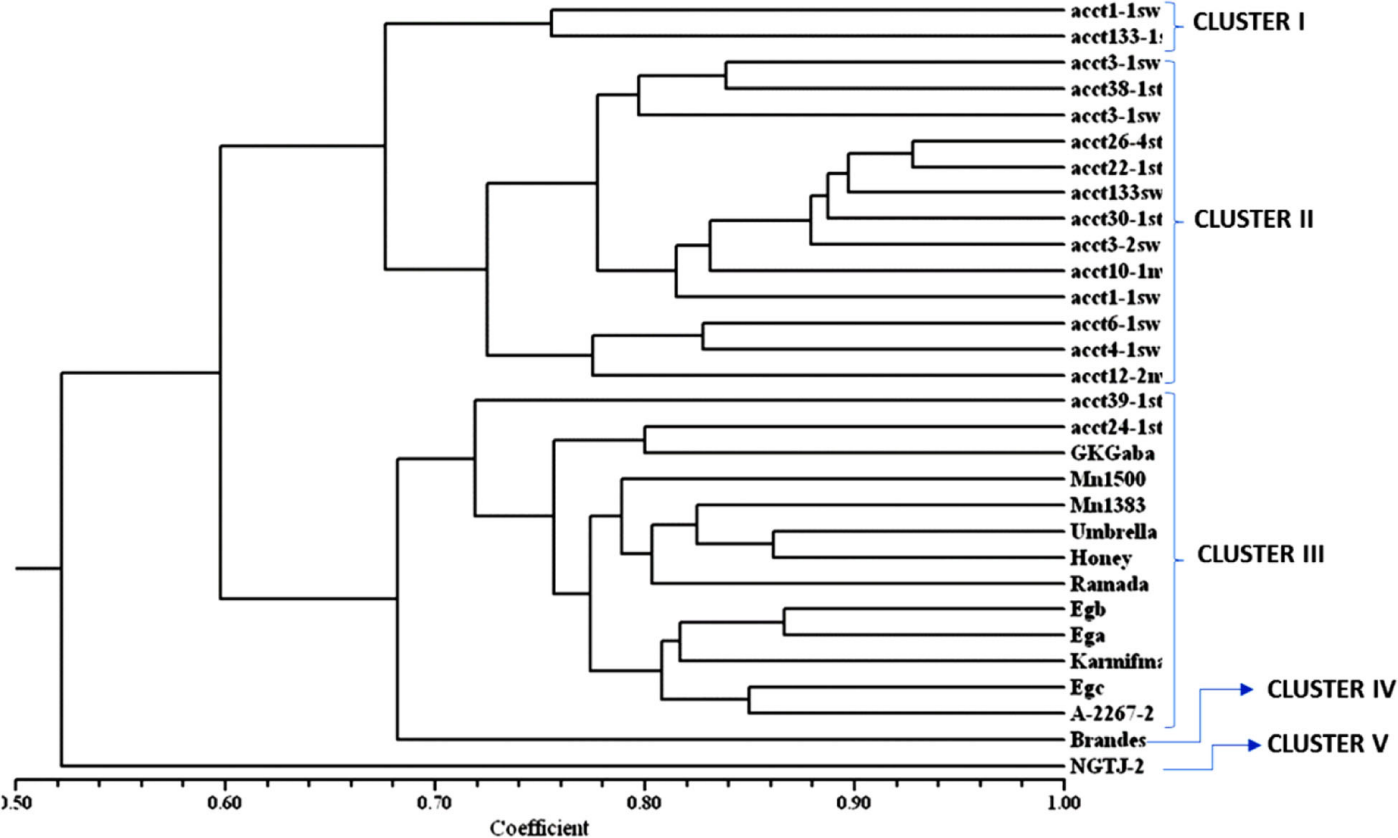
UPGMA cluster analysis based on 24 TRAP marker combinations showing similarity among 30 sweet sorghum based on similarity index

Cluster analysis performed on the thirty sweet sorghum genotypes based on similarity matrix using the unweighted pair group method with arithmetic mean (UPGMA). The genotypes were discriminated into five clusters at 0.72 coefficient of similarity (Fig. 2). Cluster I comprised two genotypes that were obtained from the same locality while cluster II and cluster III were the larger clusters, each of them containing thirteen genotypes. Clusters IV and V were found to contain one genotype each that is distinctly different from all the other genotypes. The second cluster comprised of genotypes entirely of Ethiopian origin collected in the adjoining areas indicated by the letters at the end of each genotype. All the Egyptian cultivars were found to fall in the third cluster except two Ethiopian genotypes indicating the evolutionary relatedness existing among the genotypes.

### Principal coordinate analysis

**Fig. 3:**
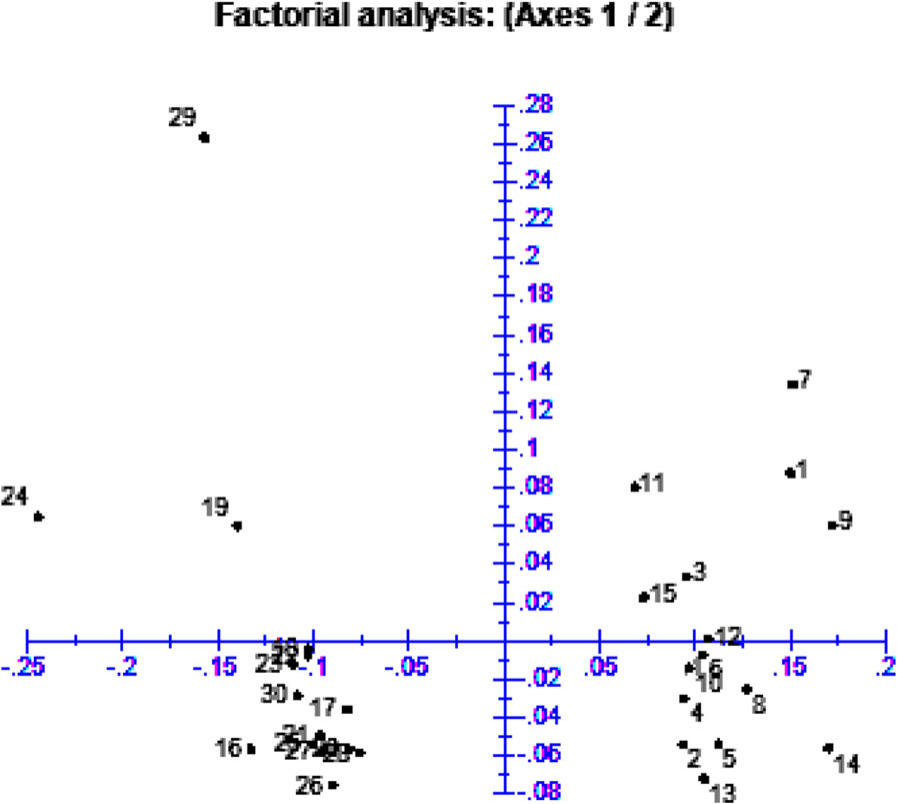
Principal coordinate analysis of 30 sweet sorghum genotypes using 24 TRAP marker combinations using the dissimilarity distance matrix

The principal coordinate analysis based on the dissimilarity matrix ordinated the genotypes into distinct classes. The pattern of the distribution of the genotypes was quite similar to the finding in the cluster analysis (Fig. 3). The three principal coordinate axes (eigenvalue > 1) were found to explain 73.97% of the total variation existing among the 30 sweet sorghum genotypes.

## Discussion

**Fig. 4:**
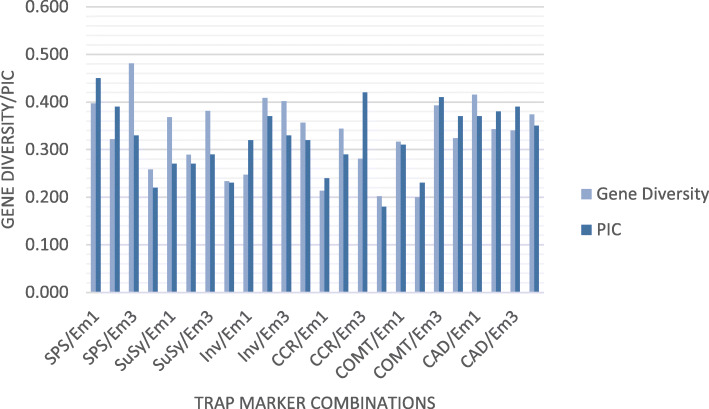
Gene diversity and polymorphic information content in 30 sweet sorghum genotypes

**Fig. 5:**
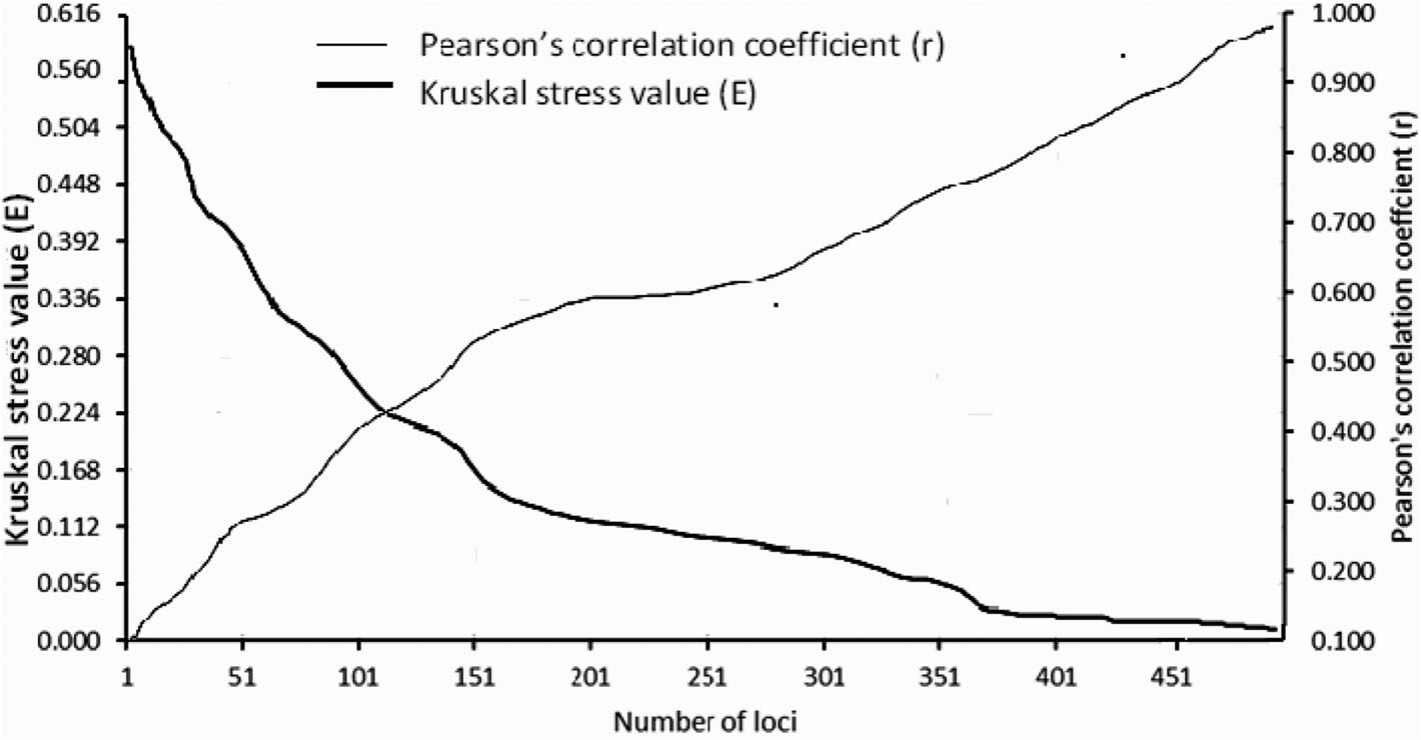
Bootstrap analysis of 24 TRAP marker combinations based on Nei’s genetic distance

This study evaluated the efficiency and informativeness of TRAP markers designed using two important biofuel related gene sequences (lignin and sucrose) to investigate the genetic diversity of germplasm resources in sweet sorghum. The results of the TRAP marker system displayed a high level of efficiency in terms of all marker attributes evaluated in comparison to other studied markers in different crops as well as in sweet sorghum. A similar result was reported with a high level of polymorphism percentage for COMT and relatively low for CAD genes on *Saccharum* complex comprising *Miscanthus*, *Erianthus*, *Saccharum spontaneum*, *Saccharum robustum*, and *Saccharum officinarum* cultivars [30]. The percentage of polymorphism for sucrose-related genes ranged between 60 (SPS) and 80% (SuSy) which is partially in agreement with that reported in sugarcane with respect to these genes [44]. These proportions signify the fact that there is an ample amount of diversity in the test materials for the target genes. The PIC values found in the present study were ranged from 0.18 to 0.42 with lignin-related genes and from 0.22 to 0.45 with sucrose-related genes. These values were wider than those obtained in the guarana plant which ranged from 0.29 to 0.37 [28] while it was comparable to the result found in sugarcane that ranged from 0.2 to 0.4 with an average of 0.3 [32]. The PIC measures the discriminatory power of a marker system where the theoretical maximum PIC value for a dominant marker is 0.5 [34]. An average PIC value of 0.32 found in the present study is also in agreement with the value found in sweet sorghum using AFLP markers [17] and in grain sorghum using SSR markers [45]. These results depicting the fact that TRAP markers can be used as a useful tool to study genetic variability in sweet sorghum. The examined TRAP marker combinations were more effective in some parameters like resolving power than a study that compared two marker systems RAPD (PIC = 0.25, H = 0.4, PP = 94.2, MI = 3.94, and RP = 4.24) and ISSR (PIC = 0.24, H = 0.38, PP = 920, MI = 3.53, and RP = 3.94) on *Justicia adhatoda* L. [46]. The bootstrap analysis confirmed that this marker system was able to generate a huge number of amplified fragments more than the number of loci estimated in order to precisely study genetic diversity. These results were similar to that reported on the *Saccharum* family which 324 markers were sufficient to explain the genetic diversity with coefficient variation of 0.5 [32], although the different techniques are used. The average gene diversity estimated by this marker system for lignin-based marker (0.374) is a relatively higher than that found for the sucrose-based markers (0.345). This finding was in contrast with that reported on sugarcane that represented 0.302 and 0.324 for genes involved in sugar and lignin metabolism, respectively [47]. These differences could be attributed to different numbers of primers and to the different genotypes used. Collectively, the TRAP marker combinations successfully assessed genetic variation among the 30 sweet sorghum cultivars and therefore, it could be potentially used in broadening the estimation tools of genetic diversity of this crop and in the breeding programs for developing promising cultivars with low lignin and high sugar content in the biofuel purposes (Figs. 4 and 5).

## Conclusions

Exploring TRAP markers as an alternative tool for assessing genetic diversity in sorghum provides opportunity for plant breeders to select potential parents with specific traits. The evaluated TRAP marker system was effective to study genetic variation in sweet sorghum genotypes. Consequently, the TRAP marker could be used as a potential marker system in expanding the characterization of genetic variability to assist breeding programs with lignin and sucrose-related genes in improving sweet sorghum crops.

## Data Availability

Not applicable

## Abbreviations

PCR: Polymerase chain reaction
PIC: Polymorphic Information Content
RP: Resolving power
EMR: Effective multiplex ratio
MI: Marker index
H: Shannon diversity index
NCBI: National Center for Biotechnology Information
EST: Expressed sequence tag
T_m_: Melting temperature
TBE: Tris borate-EDTA
UPGMA: Unweighted pair group method with arithmetic mean
SSR: Simple sequence repeat
*AFLP*: Amplified Fragment Length Polymorphism
RAPD: Random Amplification of Polymorphic DNA
SNP: Single Nucleotide Polymorphism
